# Rational Design of Self-Healing Hydrogel with High Mechanical Strength and Self-Healing Efficiency: A Short Review

**DOI:** 10.3390/gels11100807

**Published:** 2025-10-08

**Authors:** Xiaogang Yu, Jinxin Huang, Fang Yang, Jinbo Li

**Affiliations:** 1 Xinyu Key Laboratory of Materials Technology and Application for Intelligent Manufacturing, School of Mechanical and Electrical Engineering, Xinyu University, Xinyu 338004, China; 2 Xi’an Aerospace Composites Research Institute, Xi’an 710018, China

**Keywords:** self-healing hydrogel, self-healing mechanism, mechanical strength, self-healing efficiency

## Abstract

Self-healing hydrogels, a novel class of “smart” hydrogels, possess the ability to autonomously restore their network structure and mechanical properties following damage through the reconnection of a fractured three-dimensional network via reversible interactions. This characteristic enhances their safety and durability, exhibiting significant potential in biomedicine. The key determinants of self-healing hydrogels are their mechanical strength and healing efficiency. Ideally, these hydrogels exhibit both high mechanical strength and good healing efficiency. Nevertheless, an inverse relationship between the mechanical strength and self-healing efficiency of self-healing hydrogels typically exists. Thus, research is currently focused on the development of self-healing hydrogels that combine good biocompatibility, high mechanical strength, and good self-healing efficiency. This review focuses on the research progress that is being made regarding the mechanical properties and self-healing capabilities of self-healing hydrogels, where we aim to achieve a balance between self-healing performance and mechanical strength. We outline the evaluation methods for assessing self-healing performance, followed by providing a summary of recent advancements in the mechanical strength and self-healing efficiency of external-stimulus-triggered self-healing hydrogels and autonomous self-healing hydrogels. Finally, we address the challenges and prospects for the future development of self-healing hydrogels.

## 1. Introduction

**Figure 1:**
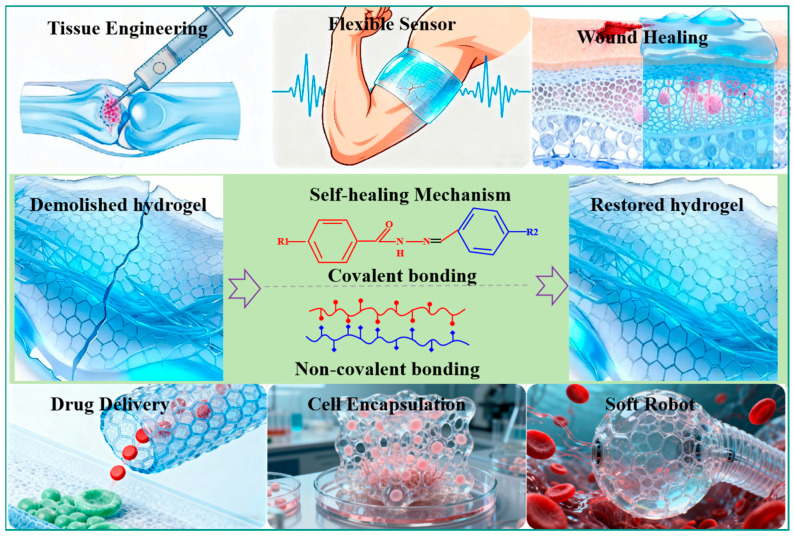
Potential applications of self-healing hydrogels in biomedicine.

Hydrogels are hydrophilic three-dimensional polymer network-structured gels formed through physical or chemical crosslinking [1]. They offer advantages such as tunable mechanical strength [2], sensitive stimulus response [3], high biocompatibility [4], and strong bionic properties [5]. Due to their high water content (50–90%), hydrogels exhibit hydration levels akin to the extracellular matrix (ECM) [6]. Their porous structure facilitates the transport of nutrients and oxygen, making them conducive to cell culture [7]. These attributes make hydrogels promising for various biomedical applications, including tissue engineering [8,9], flexible sensors [10], wound healing [11], drug delivery [12], cell encapsulation [13], and soft robots [14], as outlined in Figure 1. The statistical results show that hydrogels accounted for 50% of the global synthetic biomaterials market in 2018 [15]. Despite their numerous advantages, the rigid network of hydrogels may be susceptible to damage when applied in dynamic and load-bearing tissue environments in living organisms. This susceptibility can lead to irreversible chemical bond breakage and permanent damage, ultimately resulting in the failure of hydrogel materials. Therefore, conventional permanently crosslinked hydrogels face significant limitations in terms of biomedical applications, as the use of fragile hydrogels in living organisms not only escalates replacement costs but also poses safety risks [16]. For example, damaged hydrogels can easily enter bodily fluids, heightening the risk of inflammation [17]; compromised drug-loaded hydrogels may prematurely release internal drugs, impacting therapeutic efficacy [18]. As carriers of embedded cells, rapid cell migration-induced interface pulling can compromise the structural integrity of the hydrogel matrix, causing cells to migrate from the intended tissue site to the periphery, ultimately resulting in cell apoptosis due to an ischemic environment [18]. To address these challenges, researchers have developed hydrogels with self-healing capabilities, known as self-healing hydrogels, inspired by the nature healing processes observed in biological tissues [19]. A pioneering study performed by Toohey et al. in 2007 demonstrated the repeated self-healing of cracks in a polymer coating by designing a bio-inspired coating substrate with a three-dimensional microvascular network embedded in the substrate to deliver healing agents to the cracks [20]. Since then, self-healing hydrogels have attracted increased research interest [21].

Hydrogels’ self-healing process involves reconstructing the damaged network structure, which is typically mediated by the reversible interactions among the polymer chains. Thus, hydrogels’ self-healing capability relies on the mobility of polymer chains, which enables the re-establishment of reversible interactions. However, increased mobility of polymer chains compromises the stability of the hydrogel structure, consequently diminishing its mechanical properties. The mechanical strength of self-healing hydrogels refers to their capacity to withstand deformation and failure under external forces, which is typically assessed through tensile strength. The mechanical strength of hydrogels is influenced by factors such as polymer content, crosslinking structure, and crosslinking density, offering a broad range of adjustability. For example, elevating crosslinking density can shift the hydrogel from a liquid-like state to a solid gel state, markedly enhancing its mechanical strength while reducing the proportion of mobile phase, thereby impeding polymer the diffusion of polymer chains and reformation of reversible interactions. Thus, external stimulation is often necessary to enhance the self-healing properties of hydrogels. In essence, the mechanical strength of hydrogels hinges on the stability of their structure, whereas their self-healing performances necessitate a flow phase, presenting a challenge in terms of developing self-healing hydrogels that possess both high strength and good self-healing performance.

In this review, we aim to outline the research progress that has been made in terms of self-healing hydrogels with high mechanical properties and good self-healing efficiency, emphasizing the balance between self-healing performance and mechanical strength. We first outline the assessment methods for evaluating self-healing performance, including the observational method, dynamic self-healing performance test, and static self-healing performance test. Then, based on the requirement of external stimulation during the self-healing process, self-healing hydrogels are categorized into two types, namely external-stimulus-triggered self-healing hydrogels and autonomous self-healing hydrogels, and we review recent advancements in their mechanical properties and self-healing efficiencies. Finally, we discuss challenges and prospects for the future development of self-healing hydrogels.

## 2. Evaluation Method of Self-Healing Performance

Self-healing performance refers to a hydrogel’s capacity to restore its original network structure and mechanical strength following damage or fracture. The primary assessment methods used to evaluate the self-healing performance of hydrogels currently encompass the observational method, dynamic self-healing performance test, and static self-healing performance test, as detailed in Table 1.

### 2.1. Observational Method

The observational method is a qualitative assessment technique that is used to observe the healing process of damaged hydrogel cracks either through direct visual inspection or through various microscopic imaging modalities, such as confocal microscopy, scanning electron microscopy, and scanning electrochemical microscopy. For example, Zhang et al. [22] created a 0.9 cm diameter hole in Rhodamine B-dyed hydrogel and monitored the change in the hole’s diameter visually in order to characterize the self-healing capabilities of the hydrogel. Jiang et al. [23] cut two heart-shaped hydrogels that were dyed different colors in half. The two halves of the hydrogels, each displaying a unique color, were subsequently brought into contact at the cut edges and left to rest naturally to evaluate the hydrogel’s self-healing ability. Wei et al. [24] employed optical and electrochemical microscopy to simultaneously record the healing progression of a 2 mm scratch on the hydrogel surface, evaluating its self-healing performance. Compared with naked-eye observations, microscopic imaging technology offers a more detailed depiction of the hydrogel’s healing process on a microscopic scale, providing a comprehensive evaluation of its self-healing properties. In essence, the observational method offers a simple and direct approach for swiftly assessing the self-healing potential of hydrogels. However, it has potential biases and is typically used for qualitative characterization of self-healing performance; it is not applicable to hydrogels that require a quantitative evaluation of the overall self-healing properties.

### 2.2. Dynamic Self-Healing Performance Test

**Figure 2:**
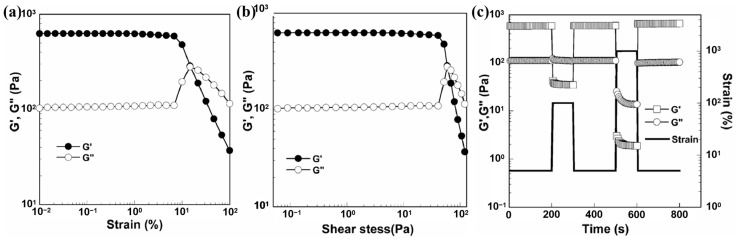
Rheology experiments: (**a**) shear strain scan; (**b**) shear stress scan; and (**c**) time scan. The vertical axis on the right is the shear strain [25] (Reprinted from Rao, Z.; Inoue, M.; Matsuda, M.; Taguchi, T. Quick Self-Healing and Thermo-Reversible Liposome Gel. *Colloid*. *Surface*. *B* **2010**, *82*, 196–202, Copyright (2011), with permission from Elsevier).

The dynamic self-healing performance of hydrogels is commonly assessed by performing dynamic rheological tests. Initially, a strain amplitude oscillation test is conducted to determine the storage modulus (G′) and loss modulus (G″) of the hydrogel, identifying the critical strain point at which the hydrogel network is disrupted. Below this critical point, where G′ exceeds G″ and both of them remain basically unchanged, the hydrogel exhibits linear viscoelastic behavior. Beyond the critical strain point, the 3D network of the hydrogel starts to collapse, leading to a decrease in G′ and changes in G″ (decrease or increase). The intersection of G′ and G″ signifies the transition of the hydrogel from a gel state to a viscous sol state. Subsequently, an alternate step strain sweep test is performed to evaluate the dynamic self-healing performance of the hydrogel. This involves alternately applying small and large shear strains (well below or above the critical strain point) alternately to the hydrogel, monitoring the recovery of G′ and G″ during cyclic loading–unloading to assess the healing state of the internal structure. Rao et al. [25] examined the self-healing properties of self-assembled liposome gels using rheological tests, as shown in Figure 2. Shear strain and shear stress scanning were initially conducted to determine the critical strain (stress) point for disrupting the hydrogel network, as shown in Figure 2a,b. The results suggested that the hydrogel exhibits linear viscoelastic behavior within the critical strain point of 10% and critical stress point of 52 Pa, with subsequent collapse of the hydrogel network beyond these critical values. Then, the alternating step shear strain experiment results depicted in Figure 2c revealed distinct behaviors of the hydrogel under varying shear conditions, as depicted in Figure 2c. At a low shear strain of 5%, G′ significantly surpassed G″, suggesting that the hydrogel maintained its gel state. Conversely, at a shear strain of 100%, the hydrogel network suffered substantial damage, resulting in a marked decrease in G′ with a minimal change in G″, indicating a shift towards a sol state. Upon reverting to a low shear strain of 5%, both G′ and G″ promptly recovered, showcasing the hydrogel’s self-healing capability. Notably, at a shear strain of 1000%, G″ exceeded G′, signifying completely destruction of the hydrogel network and its transformation into a sol state. Nevertheless, upon returning to a shear strain of 5%, both G′ and G″ swiftly recovered once more, underscoring the hydrogel’s remarkable self-healing performance even under extreme shear strain of 1000%.

In essence, the self-healing capabilities of hydrogels can be demonstrated with the use of dynamic rheological tests as they elucidate their viscoelastic behavior in response to damage and the subsequent restoration of internal crosslinks [26]. Nonetheless, this approach lacks the ability to comprehensively assess the overall mechanical characteristics of healed hydrogels, rendering it inadequate for hydrogels necessitating precise mechanical performance.

### 2.3. Static Self-Healing Performance Test

The static self-healing performance of hydrogels is commonly assessed through the use of uniaxial tensile tests. Initially, the original hydrogel specimen undergoes stretching until tensile stress–strain data fail to be record, from which the tensile strength (*σ*_0_) and break elongation (*η*_0_) of the original hydrogel specimen are determined. Subsequently, the original hydrogel specimen is bisected at its midpoint using a scalpel. The two resulting pieces are placed horizontally in contact with each other, either with a simple touch of the cut surfaces or with the addition of external stimuli to facilitate the self-healing process of the hydrogel for a specified duration. Following this, the regenerated sample is subjected to identical tensile experiments to record the tensile stress–strain data for determining the tensile strength (*σ*_h_) and break elongation (*η*_h_) of the healed hydrogel. The healing efficiency is quantified as the ratio of the tensile strength (or break elongation) of the healed hydrogel to that of the original hydrogel, as depicted in Formulas (1) and (2) [27]

(1) $$\eta_{\sigma }=\frac{\sigma_{h}}{\sigma_{o}}\times 100\%$$

(2) $$\eta_{\varepsilon }=\frac{\varepsilon_{h}}{\varepsilon_{o}}\times 100\%$$

**Figure 3:**
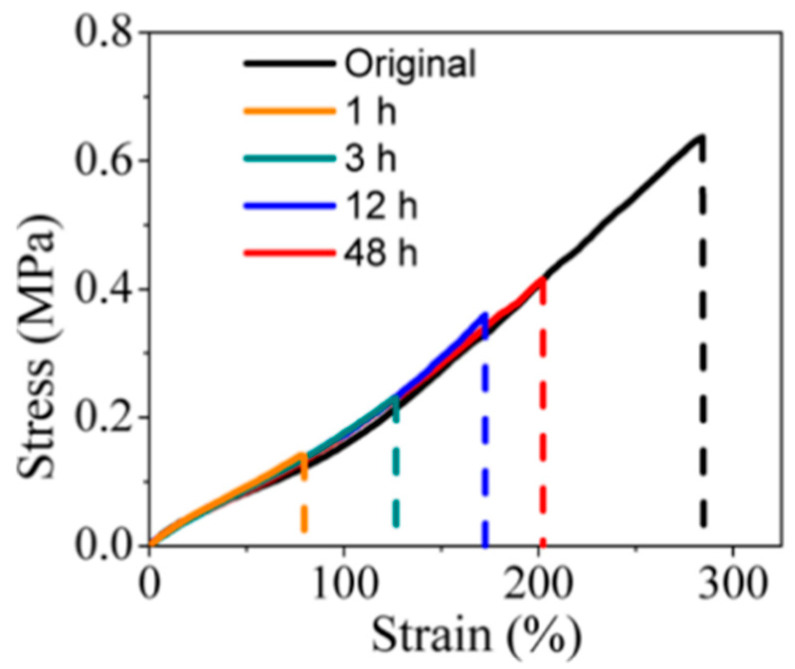
Tensile stress–strain curves of the original and self-healed PVA/PEG hydrogels at various healing times [2] (Reprinted adapted with permission from Guo, L.; Zhang, H.; Fortin, D.; Xia, H.; Zhao, Y. Poly(Vinyl Alcohol)–Poly(Ethylene Glycol) Double-Network Hydrogel: A General Approach to Shape Memory and Self-Healing Functionalities. *Langmuir* **2015**, *31*, 11709–11716. Copyright 2015 American Chemical Society).

Li et al. [2] investigated the self-healing properties of PVA/PEG hydrogels using tensile tests, as shown in Figure 3. They obtained the tensile strengths of the original and healed hydrogels via static tensile tests and calculated the self-healing efficiencies of the hydrogel in 1 h, 3 h, 12 h, and 48 h using Formula (1).

The static self-healing performance test serves as a quantitative assessment method that evaluates both the self-healing properties and the overall mechanical strength of hydrogels before and after healing. This method is particularly suitable for use in hydrogels with stringent mechanical performance requirements. Typically, a combination of the three aforementioned self-healing evaluation methods is employed to systematically assess the self-healing properties of hydrogels.

## 3. Classification of Self-Healing Hydrogel

**Figure 4:**
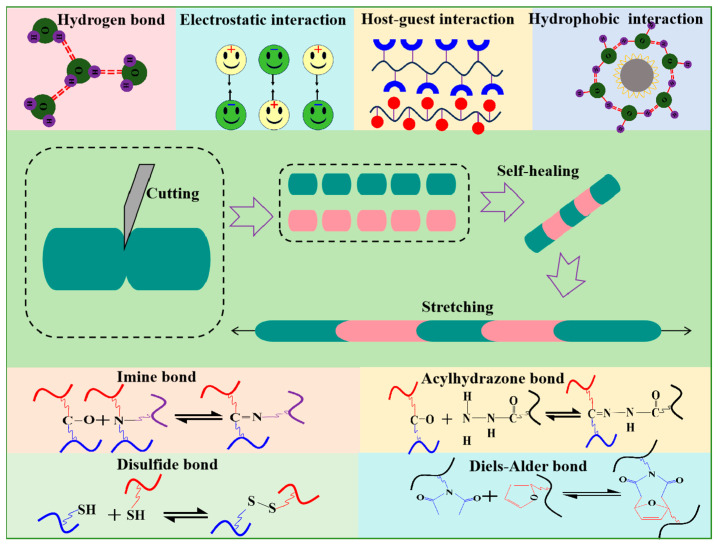
Self-healing mechanism of hydrogel.

The self-healing mechanism of hydrogels involves a dynamic equilibrium process characterized by the dissociation and recombination of the hydrogel network through reversible interactions. These reversible interactions can be broadly categorized into two groups: dynamic covalent bonds and non-covalent bonds, as shown in Figure 4. A covalent bond is a stable chemical linkage formed by two or more atoms sharing their outer electrons, resulting in a strong interaction through the sharing of electrons, while a dynamic covalent bond is a reversible covalent bond that combines the stability of covalent bonds with the reversibility of non-covalent bonds, exhibiting a strong degree of dynamics [28]. When subjected to specific external stimuli such as heat, light, force, and pH, the dynamic covalent bond can be swiftly reformed after breaking, returning to its original state and demonstrating the high stability characteristic of conventional covalent bonds. There are various types of dynamic covalent bonds, such as imine bonds, acylhydrazone bonds, disulfide bonds, and Diels–Alder bonds [15]. A non-covalent bond is a stable chemical structure formed through attractions between positive and negative charges, rather than electron sharing. Non-covalent bonds, including hydrogen bond, hydrophobic interaction, electrostatic interaction, and host-guest interaction, are reversible in nature. In contrast to dynamic covalent bonds, non-covalent bonds exhibit less bond energy and weaker stability but respond more sensitively to external stimuli such as pH and temperature, and they achieve dynamic equilibrium faster.

Based on the requirement of external stimuli to expedite the self-healing process of hydrogels, self-healing hydrogels can be classified into two categories: external-stimulus-triggered self-healing hydrogels and autonomous self-healing hydrogels. External-stimulus-triggered self-healing hydrogels necessitate external stimulation for damage repair, while autonomous self-healing hydrogels can spontaneously recover from damage without external intervention.

### 3.1. External-Stimulus-Triggered Self-Healing Hydrogels

Hydrogels’ self-healing capability is attributed to the mobility of polymer chains, which facilitates the reconstruction of reversible interactions between these chains. Essentially, the mobility of polymer chains and reversible interactions are crucial prerequisites for the self-healing ability of hydrogels. However, when hydrogels exhibit high mechanical strength, their bond energy or crosslink density increases, impeding the mobility of polymer chains. This restricted mobility subsequently inhibits the diffusion of polymer chains, thereby diminishing the self-healing efficacy of the hydrogel. Consequently, there is typically an inverse correlation between the mechanical strength and self-healing capabilities of self-healing hydrogels; high-strength hydrogels often exhibit limited self-healing efficiency under natural conditions. The key to enhancing the self-healing performance of high-strength hydrogels lies in expediting the mobility of polymer chains at the fracture interface, thereby increasing the likelihood of reconstructing reversible interactions. External stimuli serve as an effective strategy to stimulate or hasten the flow of polymer chains, thereby facilitating the self-healing of high-strength hydrogels. Common external stimuli encompass techniques such as high-temperature heating, near-infrared light irradiation, pH modulation, and the use of external repair agents.

High-temperature heating can enhance polymer chain mobility, promoting the establishment of reversible interactions between polymer chains. This process facilitates the reconstruction of the hydrogel network, enabling rapid and efficient self-healing of high-strength hydrogels. Feng et al. [29] developed a self-healing hydrogel of PDA-PNAGA-GO, leveraging the synergistic effects of multiple hydrogen bonds and π-π stacking, with a tensile strength of 0.55 MPa. In the absence of external stimuli, the fractured hydrogel exhibited limited healing even after 12 h. However, when subjected to high temperatures (90 °C), the fractured hydrogel can be mended within 10 min, achieving an approximately 80% healing efficiency.

Near-infrared (NIR) irradiation promotes the self-healing of hydrogels through a mechanism akin to high-temperature heating. This process involves the conversion of near-infrared light to heat energy using efficient photothermal conversion material within the hydrogel. The resulting heat promotes the mobility of polymer chains and the reconstruction of reversible interactions, thereby enabling the self-healing of the hydrogel [30,31,32,33]. In contrast to high-temperature heating, the temperature of NIR light irradiation can be quantitatively adjusted through modulation of the light source intensity, irradiation duration, and photothermal conversion material concentration, making it more efficient [34]. This targeted, highly efficient, and remotely controlled approach holds significant promise for practical applications in self-healing hydrogels [35,36]. Han et al. [32] developed a self-healing hydrogel by combining PDA nanoparticles with NIPAM using the photothermal conversion properties of PDA nanoparticles. The resulting PDA/NIPAM hydrogel exhibited enhanced healing capabilities upon NIR exposure. To assess the impact of PDA nanoparticles’ concentration on the hydrogel’s self-healing efficiency, different formulations were tested, including pure NIPAM hydrogel, PDA/NIPAM hydrogel with 0.15 wt.% PDA nanoparticles, and PDA/NIPAM hydrogel with 0.8 wt.% PDA nanoparticles. The results demonstrated that under NIR irradiation, the PDA/NIPAM hydrogel with 0.8 wt.% PDA nanoparticles exhibited an effective self-healing capability, whereas the other hydrogels showed limited self-healing potential. Xu et al. [37] applied Sb_2_S_3_ as a photothermal agent in a self-healing hydrogel, and it achieved nearly 100% self-healing from damage within 90 s under NIR irradiation.

The pH-dependent reversibility of interactions, such as the acylhydrazone and borate ester bonds, is pivotal to the self-healing mechanism of hydrogels [38]. Modulating the pH value can trigger the self-repair process of hydrogels containing these reversible bonds. Cong et al. [39] developed a double-network hydrogel of GO/PAACA utilizing GO nanosheets and calcium ions as dual crosslinkers. The self-healing ability of the developed GO/PAACA hydrogel exhibited significant pH sensitivity. Specifically, when the pH is below the dissociation coefficient pKa value (pKa = 4.3), the protonated carboxyl group of the PAACA side chain established strong hydrogen bonds with other polar groups, facilitating effective self-repair. Conversely, at a pH value above the pKa, deprotonation weakened the hydrogen bonds due to electrostatic repulsion of the carboxyl group, impairing the self-healing capability of the hydrogel.

External agents can also stimulate or accelerate the self-healing capabilities of hydrogels. Common repair agents include water, THF, and SDS/NaCl solution [40]. Among these agents, water holds significant potential due to its non-toxicity to human tissues and abundance in the body [41,42]. The introduction of water at the fracture interface of a hydrogel can promote polymer chain diffusion and reversible interactions, facilitating network reconstruction and enhancing self-healing efficiency [43]. Based on electrostatic interactions and hydrogen bonds, Pan et al. [42] developed PAM-DAC-GO hydrogel. To evaluate its self-healing properties, they bisected the hydrogel, brought the two sections into contact, added a water droplet at the junction, and allowed it to rest at room temperature. The results showed that the fractured hydrogel underwent automatic healing within a few hours, allowing it to stretch up to about 500% strain post-repair. Self-healing experiments demonstrated that without water, the hydrogel exhibited limited self-healing of 45.6%. In contrast, the addition of water resulted in a significantly enhanced self-healing efficiency of 92.3%.

External stimuli can promote or enhance the fluidity of molecular chains, addressing the issue of poor fluidity in high-strength hydrogels and thereby improving their self-healing capabilities. Currently, the tensile strength of externally stimulated self-healing hydrogels with self-healing efficiency surpasses 85% and can reach levels in the MPa range [44,45]. Despite their high mechanical strength, the use of external stimuli like high-temperature heating, NIR irradiation, pH regulation, and repair agents presents limitations that hinder their application in biomedicine. For example, high-temperature heating may cause irreversible damage to biological tissues. Furthermore, NIR irradiation has limited tissue penetration depth (<3 cm), pH regulation requires harsh pH conditions unsuitable for many biological tissues, and the use of repair agents is often impractical for implanted hydrogels. To overcome these limitations, researchers are increasingly focusing on developing high-strength autonomous self-healing hydrogels.

### 3.2. Autonomous Self-Healing Hydrogels

Autonomous self-healing hydrogels are capable of spontaneously repairing damage ranging from the microscopic to the macroscopic scale without any external stimuli. The mechanical properties of these hydrogels after repair typically mirror those of the original material either fully or partially. Consequently, autonomous self-healing hydrogels offer green and safer advantages for biomedical applications, garnering significant researcher attention.

Reversible interactions, such as metal ion–catechol coordination bonds [46,47] and borate ion–catechol coordination bonds [48], are frequently utilized in the development of high-strength autonomous self-healing hydrogels due to their strong bond strength. Studies on the single-molecule stretching of iron ion–catechol coordination and zinc ion–catechol bonding suggest that the fracture forces of metal ion–catechol coordination bonds are only slightly lower than those of covalent bonds under similar loading conditions, significantly surpassing non-covalent bonds like hydrogen bonds and π-π stacking [49]. Furthermore, crosslinking through metal ion–catechol coordination bonds offers a combination of stability that is comparable to covalent bonds and the reconstruction efficiency of non-covalent bonds, with the additional advantage of multiple potential crosslinking points (1–3 per metal ion). Consequently, autonomous self-healing hydrogels based on metal coordination bonds can possess high mechanical strength and self-healing efficiency. The tensile strengths of reported autonomous self-healing hydrogels relying on metal coordination bonds typically range from 0.3 MPa to 1.38 MPa, with self-healing efficiencies exceeding 85% [49,50,51,52]. For instance, Shao et al. [52] developed PAA-CNFs-Fe^3+^ hydrogels based on metal coordination bonds and hydrogen bonds, demonstrating a tensile strength of up to 1.38 MPa. Self-healing performance test experiments show that this hydrogel’s self-healing efficiency can reach up to 94.2% after 48 h.

Despite its high mechanical strength and good self-healing performance, the potential toxicity of metal ions in metal coordination-based hydrogels restricts their biomedical applications. For example, excessive iron ions can trigger Fenton reactions, generating reactive oxygen species that harm biomolecules and lead to methemoglobinemia [53]. Consequently, there is a need to create biocompatible self-healing hydrogels with high mechanical properties and efficient self-repair capabilities for biomedical use.

For biocompatibility to remain, non-covalent bonds other than metal coordination bonds, such as hydrogen bonds and π-π stacking, are utilized in the development of autonomous self-healing hydrogels. The reported PDA-PAM hydrogel [54] is a typical autonomous self-healing hydrogel based on hydrogen bonds and π-π stacking, whose self-healing efficiency goes up to 98% within 2 h, but its tensile strength is only 8 kPa. Increasing the number of non-covalent bonds can effectively improve the mechanical strength of autonomous self-healing hydrogels. By adjusting the pH and ammonium persulfate content of the hydrogel solution to promote dopamine polymerization, Huang et al. [55] increased the PDA concentration within PDA-PAM hydrogel to increase the number of hydrogen bonds to improve its tensile strength. The tensile strength of their prepared PDA-PAM hydrogel was enhanced to 16 kPa while maintaining a self-healing efficiency of 96% in 2 h. Due to its high density of hydroxyl groups capable of forming numerous hydrogen bonds, PVA is regarded as a highly promising material for self-healing hydrogels. Zhang et al. [56] developed a PVA hydrogel with a tensile strength of 262 kPa, whose self-healing efficiency is 72% in 48 h. Furthermore, Huang et al. [57] created a PVA-TA hydrogel by leveraging the abundant hydrogen bonds formed between PVA and TA, resulting in a tensile strength of 224 kPa and a self-healing efficiency of 87% in 2 h. Increasing the bond energy of reversible interactions can also enhance the tensile strength of autonomous self-healing hydrogels. Yu et al. [58] introduced electrostatic interactions, which possess higher bond energy than hydrogen bonds, into the PVA-TA hydrogel, thereby preparing a PVA-TA/CS hydrogel with a tensile strength of 447 kPa, and it reached 84% self-healing in 2 h.

Mechanical strength and self-healing efficiency are crucial characteristics of autonomous self-healing hydrogels; however, achieving both of these characteristics simultaneously poses challenges. This dilemma arises from the fact that autonomous self-healing hydrogels with elevated tensile strength typically exhibit increased bond energy or crosslink density, impeding molecular chain mobility and hindering polymer diffusion and crosslink network reconstruction, thereby leading to diminished self-healing efficiency [15]. The reported tensile strength of biocompatible autonomous self-healing hydrogels with good self-healing efficiency predominantly falls within the range of 8 kPa–450 kPa, as outlined in Table 2.

## 4. Conclusions and Outlook

The ability of biological tissues to regenerate spontaneously following damage has inspired the development of “smart” hydrogels with self-healing capabilities. These hydrogels can restore their three-dimensional network and mechanical properties after suffering physical damage. This advancement not only prolongs the lifespan of hydrogels but also reduces the costs associated with their repair and replacement. This is beneficial as hydrogels are widely used in tissue engineering, sustained drug release, and as biological tissue substitutes. Despite the rapid progress in self-healing hydrogel research, the translation of these findings to hydrogels’ clinical use faces challenges. An ideal self-healing hydrogel should not only possess sufficient mechanical strength but also have excellent self-healing efficiency to ensure rapid autonomous repair to damage without an external stimulus. Moreover, for hydrogels implanted into organisms, excellent biocompatibility and easily achievable self-healing are also necessary. Autonomous self-healing hydrogels, characterized by their ability to spontaneously and rapidly mend damage without external intervention, hold significant promise for biomedical applications. However, a common issue lies in the trade-off between self-healing capacity and tensile strength, resulting in reported autonomous self-healing hydrogels with suboptimal mechanical properties that fall short of meeting the requirements for biomedical use, necessitating strength akin to natural tissues such as cartilage, skin, and muscles. Overcoming this challenge to develop a biocompatible self-healing hydrogel with both sufficient mechanical strength and efficient self-healing capabilities remains a formidable task. The inherent dilemma between mechanical strength and molecular chain mobility stands out as a key obstacle in the advancement of such hydrogels. The use of double-network hydrogels, which comprise two interwoven crosslinked networks, is a promising option for developing high-mechanical-strength hydrogels. Numerous studies have focused on creating self-healing double-network hydrogels with good mechanical properties. However, the fundamental challenge of balancing mechanical strength with molecular chain mobility remains a significant barrier to the development of self-healing hydrogels. One potential strategy to address this challenge involves exploring innovative external stimulus approaches to enhance the fluidity of molecular chains in self-healing hydrogels while maintaining high mechanical strength. 

The utilization of external stimulus to modulate polymer chain mobility in high-strength biocompatible hydrogels emerges as a promising strategy for overcoming the traditional trade-off between mechanical strength and self-healing capability. This approach offers a significant advantage by enabling the manipulation of polymer chain dynamics without affecting the inherent crosslink density of the hydrogels, which is crucial for their mechanical robustness. By doing so, it offers a viable route to concurrently maintain high mechanical performance and improve self-healing efficiency. The choice of external stimulus modality is pivotal for this strategy, as it directly impacts its applicability in biomedical settings, particularly in terms of tissue compatibility and penetration depth. Among candidate stimuli, magnetic field stands out for their unique advantages in biomedical applications due to its deep tissue-penetrating capabilities and non-invasiveness. Despite these merits, the technological maturity of magnetic-responsive self-healing hydrogels remains constrained by two factors: (1) the optimization of magnetic responsiveness, which involves tuning the loading, surface modification, and spatial dispersion of magnetic nanoparticles to enhance sensitivity to magnetic field, and (2) the development of scalable fabrication strategies, such as one-step in situ polymerization, to ensure the uniform integration of magnetic nanoparticles into the polymer network without causing aggregation-induced cytotoxicity or mechanical heterogeneity. Overcoming these challenges is critical for the practical implementation of this stimulus-aided approach.

## Figures and Tables

**Table 1:** Methods used to evaluate self-healing performance.

Method	Characteristics
Observational method	Simple and fast, yet not quantitatively evaluable.
Dynamic self-healing performance test	Capable of monitoring variations in storage modulus and loss modulus, yet unable to evaluate the tensile strength.
Static self-healing performance test	Quantitatively evaluate the self-healing efficiency under 100% destruction to fully understand the recovery situation of the tensile strength.

**Table 2:** Summary of properties of biocompatible autonomous self-healing hydrogels.

Hydrogel	Tensile Strength	Self-Healing Time	Self-Healing Efficiency	Ref.
PDA-PAM	8 kPa	2 h	98%	[54]
PDA-talc-PAM	8.5 kPa	2 h	60%	[16]
PDA-PAM	16 kPa	2 h	96%	[55]
PDA-PGO-PAM	21 kPa	24 h	62%	[27]
DF-PEG	23 kPa	24 h	100%	[59]
Agarose/PVA	25 kPa	10 s	100%	[60]
β-CD-AOI_2_-A-TEG-Ad	28 kPa	1 h	63%	[61]
PNIPAM-PAM-clay	60 kPa	150 h	90%	[62]
CNF-PPy/PB	63 kPa	20 s	97%	[63]
PVA/PAA	160 kPa	12 h	37%	[64]
Zr-NC gel	195 kPa	12 h	75%	[65]
PVA-TA	224 kPa	2 h	87%	[57]
PVA	278 kPa	48 h	72%	[56]
PVA-CS/TA	447 kPa	2 h	84%	[58]

## Data Availability

No new data were created or analyzed in this study. Data sharing is not applicable to this article.

## Abbreviations

The following abbreviations are used in this manuscript:

PVA	Polyvinyl alcohol
PEG	Polyethylene glycol
PDA	Polydopamine
PNAGA	Poly N-acryloyl glycinamide
GO	Graphene oxide
NIPAM	N-isopropylacrylamide
PAACA	Poly N-acrylyl-6-aminocaproic acid
DAC	2-dimethylaminoethylacrylate
PAM	Polyacrylamide
PAA	Polyacrylic
TA	Tannic acid
CS	Chitosan
THF	Tetrahydrofuran
SDS	Sodium dodecyl sulfate
CNF	Cellulose nanofiber
